# Transseptal Approach Versus Transaortic Approach for Catheter Ablation of Left-Sided Accessory Pathways in Children

**DOI:** 10.3389/fped.2022.888029

**Published:** 2022-06-17

**Authors:** Xia Yu, Ziyan Dong, Lu Gao, Li Lin, Lang Cui, Wei Shao, Wen Yu, Zhen Zhen, Yue Yuan

**Affiliations:** Department of Cardiology, National Center for Children’s Health, Beijing Children’s Hospital, Capital Medical University, Beijing, China

**Keywords:** transseptal approach, aortic approach, left-sided accessory pathway, catheter ablation, pediatrics

## Abstract

**Background:**

Catheter ablation is recommended to eradicate supraventricular tachycardia caused by left-sided accessory pathways (APs) in children. This study aims to compare the safety and efficacy of the transseptal approach (TS) and aortic approach (TA) for catheter ablation of left-sided APs in a pediatric cohort.

**Methods:**

Patients < 18 years of age with left-sided APs who had undergone ablation at Beijing Children’s Hospital between 13 January 2018 and 7 January 2020 were included and randomly categorized into either TS or TA group (follow-up for 22 months). In all, 60 patients in TS group and 41 patients in TA group were included in this study. Principal endpoints were success rate, recurrence rate, complications, procedure time, and radiation dose.

**Results:**

For TS group versus TA group, success rate was observed in 100 versus 97.56% (*p* = 0.402). The procedure time was 27.0 (32.0–23.0) versus 29.0 (38.0–24.5) min (*p* = 0.092). The rate of success or the procedure time was similar, but for the patients with Aps located in left posterior septum (LPS) or left posterior lateral (LPL), the TS group had a shorter procedure time compared with TA group (*p* < 0.01). The radiation dose was 28.0 (20.0–41.75) versus 0 mGy (*p* < 0.001). After successful ablation, no recurrence and complication were observed in either group.

**Conclusion:**

Both TS and TA for catheter ablation of left-sided Aps were shown to be safe and effective in children. Zero radiation and ease of mastery make TA the preferred choice. TS is recommended to be used by properly trained medical professionals, especially for patient with AP localized in the LPL or LPS. However, TS is a good alternative where patients have aortic lesions or when TA fails.

## Introduction

Catheter ablation is recommended to eradicate supraventricular tachycardia caused by left-sided accessory pathways (Aps) in children, which can be performed by a transseptal approach (TS) or an aortic approach (TA). A meta-analysis of adults suggested that TS provides higher acute success and lower incidence of complications; however, no large randomized trials have been published to confirm these findings (1). There have also been no studies on children. Therefore, the purpose of this study was to compare the safety and efficacy of transseptal approach (TS) and aortic approach (TA) for catheter ablation of left-sided Aps in children.

## Materials and Methods

### Study Subjects

This retrospective cohort study was performed at Beijing Children’s Hospital (BCH). All children and adolescents < 18 years of age with left-sided Aps, who were scheduled for catheter ablation in BCH from 13 January 2018, to 7 January 2020, were included in this study. Data from primary and secondary ablation patients were assessed separately to avoid bias.

*Inclusion criteria*: 1. Children diagnosed paroxysmal supraventricular tachycardia by electrocardiogram (ECG), echocardiography, physical examination, and tachyarrhythmia recurrence 2 times or more. 2. Electrophysiological examination was used to confirm that the Aps are located on the left side. 3. Drug treatment was ineffective and could not be tolerated long-term, or parents ask for surgical treatment. 4. All guardians of children signed informed consent to comply with the 2013 Declaration of Helsinki.

*Exclusion criteria*: 1. The patient had surgical or anesthesia-related contraindications. 2. Patient had serious structural heart disease (leading to decreased cardiac function) or other systemic diseases. 3. Patient did not have spontaneous or induced arrhythmia during catheter ablation.

## Study Method

### Preoperative Preparation

All children discontinued antiarrhythmic drugs for 3 half-lives before electrophysiological examination, and all fasted for more than 8 h prior to surgery.

### Surgical Process

*Cardiac electrophysiological examination:* Cardiac electrophysiological examination was performed on all patients under general anesthesia, guided by Ensite NavX system (a 3-dimensional catheter navigation system). The St. Jude electrode was placed in the right ventricle (Hirschner’s bundle) and coronary sinus using the left femoral vein and right internal jugular vein approach. To delineate the position of the AP, atrioventricular reentrant tachycardia (AVRT) was initiated using programmed electrical stimulation in the atrium or ventricle.

Patients meeting cardiac electrophysiological examination criteria were included. Criteria included (1) atrial programmed stimulation could not induce atrioventricular nodal skipping; (2) ventricular stimulation showed ventricular atrium 1:1 reverse transmission and did not decrease; (3) atrial graded stimulation and atrial program stimulation-induced stable tachycardia; (4) surface ECGs were considered as atrioventricular reentrant tachycardia; (5) the AP is located on the left side. Children with double Aps and combined atrioventricular nodal reentrant tachycardia were excluded. Catheter ablation was performed by the same operator in all patients in both groups.

**FIGURE 1:**
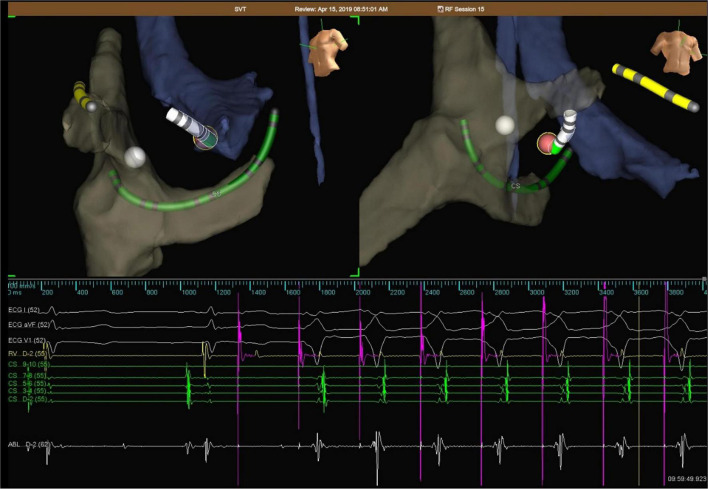
Representative electroanatomical map during ablation of left -sided AP via TA in a child. Catheters are placed by the aid of NAVX, showing a small A and a large V, having AV fusion during ventricular pacing. The white tag represents the His region, while red tag represents ablation point. AV separation indicates that ablation is effective.

*TA approach* (Figure 1): The TA approach was used in all patients of TA group. After the sheath was placed into the right femoral artery, the bipolar catheter was inserted through the sheath, advanced to the aorta, and prolapsed through the aortic valve. Under the guidance of NAVX, in combination with the surface ECG and cardiac electrophysiological examination result, the catheter was then placed at the target site, showing a small A and a large V, having AV fusion during ventricular pacing. Catheter ablation was delivered with a power of 15–20 W and a target temperature of 52–55°C under sinus rhythm. AV separation indicates that ablation is effective, and consolidation ablation was repeated 3 times, each lasting for 60 s.

**FIGURE 2:**
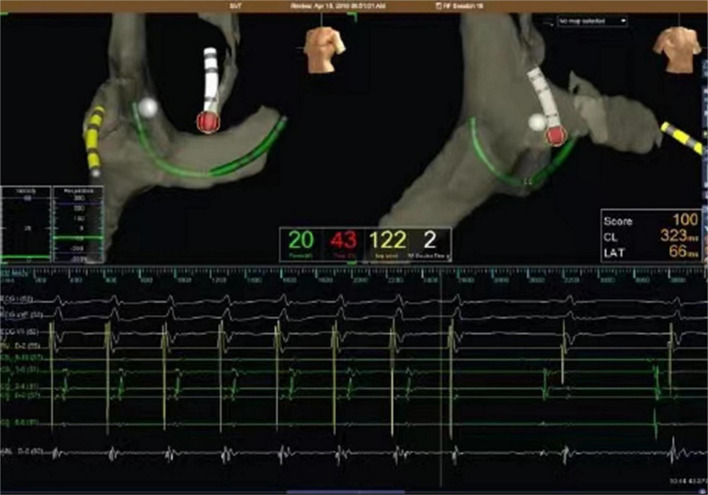
Representative electroanatomical map during ablation of left -sided AP via TS in a child. Catheters were placed under the guidance of NAVX after successful puncture, showing a small A and a large V, having AV fusion during ventricular pacing. The w hite tag represents the His region, while red tag represents ablation point. AV separation indicates that ablation is effective. AV separation indicates that ablation is effective.

*TS approach* (Figure 2): The TS approach was performed under fluoroscopic guidance, with first the guide-wire, and then, the long sheath and dilator were placed in the right femoral vein, advanced to the superior vena cava. Next, the transseptal needle was pushed to about 1 cm from the end of the dilator. The transseptal needle, dilator, and long sheath were moved to the fossa ovalis located in the posteroanterior position. When the atrial septum was punctured by a transseptal needle, a contrast agent was injected and dispersed toward the spine. The ablation electrode was placed into the left atrium under the guidance of NAVX after successful puncture, and catheter ablation was delivered with a power of 30–40 W and a target temperature of 52–55°C. The tachycardia was terminated after discharge, and consolidation ablation was repeated 3 times, each lasting for 60 s.

### Follow-Up

All patients had followed up appointments spaced at 1, 3, 6, and 12 months after discharge from the hospital (22 months in total). An ECG, Holter monitoring, and echocardiography were performed at each follow-up. Those lost to follow-up at BCH were followed up by telephone to determine whether there was disease recurrence and related complications. All follow-up data from hospital visits and telephone were used in the analysis.

### Observation Index

(a) *Success rate*: Successful catheter ablation was defined as: no tachycardia observed for 45 min, a static point of isoproterenol and repeated programmed electrical stimulation failing to induce tachycardia, and no need for antiarrhythmic drugs 1 month post-ablation. Success rate = the number of patients with successful radiofrequency ablation/total number of patients included in the study.
(b) *Recurrence rate*: Recurrence was defined as the occurrence of the same tachycardia seen before ablation or the occurrence of the original delta wave on the surface ECG in the follow-up period.
  Recurrence rate = the number of patients with recurrence/total number of patients receiving specific catheter ablation.
(c) *Complications*: Complications included vascular puncture, local hemorrhage, hematoma, infection, pneumothorax, thrombosis, embolism, complications of catheter operation (aortic regurgitation, myocardial perforation, and pericardial tamponade), complications of discharge ablation (atrioventricular block, myocardial infarction), and complications of penetrating atrial septum (pericardial effusion, pericardial tamponade). Suspected patients were examined for early identification.
(d) *Location of AP*: The location of APs was delineated in cardiac electrophysiological examination, which is divided into four groups: left lateral (LL), left posterior septum (LPS)/left posterior lateral (LPL), left anterior lateral (LAL), or left middle septum (LMS).
(e) *Procedure time*: Procedure time of TS was defined as the time taken from sending the ablation electrode to the left atrium until the termination of tachycardia, and the operation time of TA was defined as the time taken from sending the ablation electrode to the left ventricle until the termination of tachycardia. Times were recorded in all patients.
(f) *Radiation dose*: Radiation dose is defined as the total amount of radiation given from the beginning until the end of the procedure, which was recorded in all patients.

### Data Analysis

Statistical analysis was performed using SPSS 16.0 software. The continuous data of normal distribution were expressed as mean ± SD, while of non-normal distribution is expressed as the median (interquartile range). Categorical data are expressed as the quantity (percentage). Normal distribution measurement data were compared using Student’s *t*-test, while of non-normal distribution was compared using the Mann–Whitney U rank-sum test. Comparisons of categorical data were performed using chi-square and Fisher’s exact test. A two-tailed *p*-value of < 0.05 was considered significant.

## Results

### Baseline Characteristics

A total of 101 patients < 18 years of age with left-sided APs had undergone ablation at BCH, of which 60 patients were in the TS group and 41 patients in the TA group. Only 1 patient had a successful catheter ablation using TS after the failure of using TA. Patient baseline characteristics are shown in Table 1.

**TABLE 1:** Baseline characteristics of patients in TS and TA groups.

	TS (*n* = 60)	TA (*n* = 41)	*P*-value
Age (months)	130.5(150.0–90.5)	110.0 (147.5–80.0)	0.091
Male	38 (62.3%)	27 (65.9%)	0.607
Body weight (kg)	38.5 (48.0–28.25)	32.0 (46.5–23.0)	0.304
Body weight<30kg	17 (27.9%)	17 (41.5%)	0.201
Other heart problems			
Cardiomyopathy	2 (3.3%)	1 (2.4%)	0.999
Patent foramen ovale	1 (1.6%)	0	
Ventricular septal defect	0	1 (2.4%)	
The location of APs			
LL	33 (55.0%)	32 (78.0%)	0.060
LPS/LPL	12 (20.0%)	2 (4.9%)	
LAL	13 (21.7%)	6 (14.6%)	
LMS	2 (3.3%)	1 (2.4%)	

*Values are median (interquartile range) or n (%).*

*TS, transseptal approach; TA, aortic approach; LL, left lateral; LPS/LPL, left posterior septum/left posterior lateral; LAL, left anterior lateral; LMS, left middle septum.*

For TS group versus TA group, the mean age of the patients who underwent ablation was 130.5 (150.0–90.5) months versus 110.0 (147.5–80.0) months; the weight was 38.5 (48.0–28.25) versus 32.0 (46.5–23.0) kg and the number below 30 kg was 17 versus 17, 38 patients were men (63.3%) and 22 were women (36.7%) in the TS group, whereas 27 (65.9%) patients were men and 14 (34.1%) were women in the TA group. Both groups had similar age and weight (*p* = 0.091, 0.304), and there was no statistically significant difference in gender by the chi-square test (*p* = 0.607).

Other mild heart problems, such as cardiomyopathy, patent foramen ovale, ventricular septal defect, or mitral regurgitation, were recorded and proved no difference between groups (*p* = 0.999). Due to the potential risk of aortic pathology, Marfan syndrome has been identified as relative contraindication to transaortic approach. So, children with Marfan syndrome were excluded in the study.

The location of APs was an important factor that affects the procedure process. For the AP of TS group versus TA group, 33 (55.0%) versus 32 (78.0%) were localized in LL, 13 (21.7%) versus 6 (14.6%) were localized in LAL, 12 (20.0%) versus 2 (4.9%) were found to be left posterior, and 2 (3.3%) versus 1 (2.4%) were in LMS. The distribution of APs location was not significantly different between the two groups by Fisher’s exact test (*p* = 0.060).

### Success Rate, Recurrence Rate, and Complications

Success rate of TS group and TA group was 100 versus 97.62%, as 1 patient with AP localized in LPL had a successful catheter ablation using TS after failure of TA. However, the success rate was comparable in both groups (*p* = 0.406). After successful ablation, no recurrence or complication was observed in either group (Table 2).

**TABLE 2:** Ablation outcomes and follow-up results of patients in TS and TA groups.

	TS (*n* = 60)	TA (*n* = 41)	*P*-value
Success rate (%)	100	97.62	0.406
Recurrence rate (%)	0	0	0.999
Complications*	0	0	0.999
Overall procedure time (min)	27.0 (32.0–23.0)	30.0 (40.5–24.5)	0.092
LL-procedure time(min)	27 (22.5–32.5)	28.5 (24.25–38.0)	0.229
LPS/LPL-procedure time (min)*	26 (21.5–28.5)	58.5 (49.0–68.0)	0.022
LAL-procedure time(min)	31 (25–36.5)	28.0 (20.25–39.25)	0.521
LMS-procedure time(min)	29.0 (27.0–29.0)	43.0	0.667
Radiation dose (mGy)*	28.0 (20.0–41.75)	0	<0.001

*Values are median (interquartile range) or n (%).*

*The threshold for significance is from P < 0.05 to P < 0.001.

*TS, transseptal approach; TA, aortic approach; LL, left lateral; LPS/LPL, left posterior septum/left posterior lateral; LAL, left anterior lateral; LMS, left middle septum.*

### Procedure Time

Procedure time of TA group and TS group was 30.0 (24.5–40.5) and 27.0 (32.0–23.0) min, respectively, indicating that the former group procedures were slightly longer than the latter, but the difference between them was not significant (*p* = 0.092) (Table 2).

Different results occurred after considering the location of AP. Procedure times were shorter in TS group when APs were derived from LPS or LPL (*p* = 0.022). When the bypass was localized in the LL, LAL, or LMS, the operation time was comparable in both groups (*p* = 0.279, 0.521, and 0.667).

### Radiation Dose

The radiation dose of TS group and TA group was 28.0 (20.0–41.75) versus 0 mGy, respectively; thus, patients in the TA group received significantly less radiation (*p* < 0.001) (Table 2).

## Discussion

Overall, the success rate, recurrence rate, and complication rate did not differ between the TA and TS treatment groups. With TS treatment, procedure times were reduced when AP was derived from LPS or LPL, but patients also received more radiation, thus requiring a tradeoff of time versus radiation.

The TA is a common procedure to obtain access to the left heart during cardiac catheterization of left-sided APs in children. Scaglione et al. reported an ablation using TA in 44 children with the left-sided APs, guided by CARTO-3 mapping system, with fluoroscopy time of 0 min, success rate of 100%, and a recurrence rate of 16% (2). Ayabakan et al. reported 96% of success rate, 8% of recurrence rate, and 0% of serious complications of left-sided AP by TA (3). In this study, no recurrence was observed, and success rates and complication rates were comparable with previous studies (2, 3).

Transseptal approach, because of its need for skilled catheter manipulation and has potential risks of serious complications, including pericardial effusion and cardiac tamponade, is still not widely used by most cardiovascular pediatricians (4, 5). However, TS in infants and children has been described as effective and safe with low complication rates in many studies. Yoshida, Rami, and Koca also demonstrated the safety and high efficiency of the TS for ablation in children with left-sided APs weighing less than 30 kg (6–8). The success rates were 100, 98.8, and 97.8% in 43, 86, and 45 cases, with complication rates of 2.3, 0, and 2%, respectively. However, TS is reported to be a high-risk procedure in infants under 1 year of age or weighing < 5 kg with a restrictive intra-atrial septum and the need for enlargement of the intra-atrial communication (9). In this study, the successes rate was 100% with no complications in the TS group, which was comparable with previous studies.

Many medical institutions prefer TA to TS (3). Serious potential complications with TS have been reported (4, 5). Katritsis et al. documented 1.27% cardiac tamponade among 393 patients with the use of the TS (4), whereas von Alvensleben et al. reported a 1.9% pericardial effusion among pediatric patients undergoing TS for ablation (5). In contrast, TS requires special training and must be performed by experienced and superb operators. As some medical personnel are not trained for this approach, to avoid additional risk, managing left-sided APs by TA is more suitable.

Some cardiovascular pediatricians hold the opposite view. TS was considered as a better alternative to TA in the study of Zhu et al., primarily due to a lower recurrence rate (10). If the AP is obliquely distributed, the site of earliest retrograde activation recorded from the ventricular lateral annulus may not be the real ventricular insertion point of the AP. In addition, TA may increase the procedure time and injure the aortic valve during the ablation. With a smaller ascending aorta and left ventricle in children, the catheter is often ejected from the left ventricle, and repeatedly crosses are needed.

In our study, the recurrence rate was comparable in both groups, and no injury of aortic valve was found in the TA group. Further, patients in the TA group received significantly less radiation. In addition, although no major complications were found in TS group, complications were more severe in TS than in TA. Considering the simplicity of operation and the radiation dose, we recommend TA to be the first choice for ablation of left-sided APs in children. However, for professionals with advanced training, TS has advantages in some situations.

**FIGURE 3:**
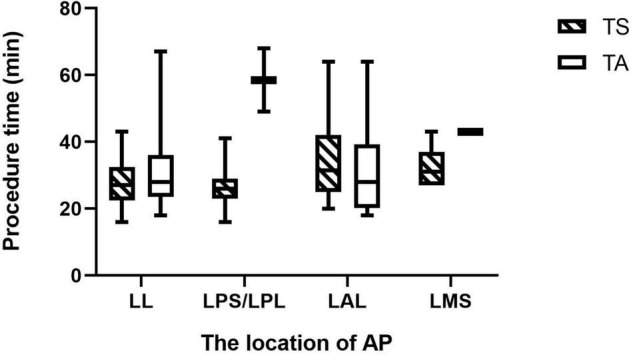
This Box-plot presents the procedure time for APs at different locations. Although the overall procedure time did not differ between the two groups, the TS group had a shorter procedure time for patients with APs located in LPS/LPL (*P* = 0.022). TS, transseptal approach; TA, aortic approach; LL, left lateral; LPS/LPL, left posterior septum/left posterior lateral; LAL, left anterior lateral; LMS, left middle septum; AP, accessory pathway.

Although the overall procedure time did not differ between the two groups, the TS group had a shorter procedure time for patients with APs located in LPS/LPL (Figure 3). Also, 1 patient with AP localized in LPL had a successful catheter ablation using TS after failure with TA. This might be attributed to the easier manipulation of the ablation catheters in the left atrium by TS when AP localized in LPS or LPL, compared to the more challenging manipulation approaching from the left ventricle across the aortic arch. Further, in TS, the stability of the catheter is improved by its passage through and fixation to the interatrial septum, allowing better contact with the mitral annulus and more effective delivery of radiofrequency. The procedure time recorded in this study, due to the different definitions and the use of 3D-electroanatomical mapping system, was shorter than that in another similar study (7).

Peripheral vascular disease is the most common cause of complications using the transaortic approach for left accessory pathway ablation in adults. Although the incidence of peripheral vascular disease is low in children, congenital heart disease involving the aorta is more common. TA approach has the limitation that it cannot be applied in patients with aortic lesions, such as aortic stenosis or aortic aneurysm. For safety, adequate assessment of vascular and cardiac conditions is particularly important before selecting an ablation approach. Although excluded during data analysis, a child with Marfan syndrome was successfully ablated in TS group. Since his echocardiography revealed a full aortic sinus, TA was contraindicated considering the possibility of aortic aneurysm. Furthermore, TS is recommended in the following situations: (1) APs localized in LPL or LPS, (2) patients with aortic lesions, and (3) after the failure of TA.

The risk of radiation exposure in ablation is well recognized for patients, especially in children. Radiation increases the lifetime risk of carcinomas and hematopoiesis abnormal (11, 12). Children are more vulnerable to this risk because they are more sensitive to radiation than adults, and there is a latent period between radiation exposure and cancer presentation (13). At present, a 3D-electroanatomical mapping system is widely used in the field of radiofrequency ablation to reduce fluoroscopy exposure and is shown to be safe and effective among patients with pediatrics (8). In this study, zero fluoroscopy was achieved in TA group using the NAVX system. However, complete 3D-electroanatomical mapping and zero-fluoroscopy approach are difficult to achieve in ablation by TS. Combined fluoroscopy for TS provides real-time images of tracheal, CS electrode, and spine which are helpful in locating the puncture site of an atrial septum. Fluoroscopy assistance is necessary to ensure safety, especially for children with cardiac and macrovascular lesions. TS may be challenging for children with structural heart diseases, such as a thick/fibrotic or aneurysmatic interatrial septum. Some additional imaging tools, such as transthoracic echocardiography (14), transesophageal echocardiography (15, 16), or intracardiac echocardiography (17), are used to improve safety and efficacy of TS in patients with difficult anatomy or small, critically ill babies.

For adults with left atrial arrhythmias, zero-fluoroscopy ablation can be achieved by accessing the left atrium through a patent foramen ovale. Michael et al. demonstrated that this approach was feasible in majority of patients, even in case of failure, total fluoroscopy time and radiation dose were very low (18). Although the smaller cardiac structure in children increases the difficulty of this approach, it can be potentially used as well. In Scaglione’s study, with the help of transesophageal echocardiography and intracardiac echocardiography, two children with left-sided AP successfully achieved zero-fluoroscopy ablation through the patent foramen ovale (2). But for children with closed foramen ovale, it is urgent to develop new technologies and methods to realize zero-fluoroscopy atrial septal puncture. Considering the higher radiation risk in children, TA is the preferred access, despite similar efficacy and safety.

## Conclusion

Both TS and TA for catheter ablation of left-sided APs were shown to be safe and effective in children. TA is the preferred access to prevent radiation exposure, despite similar efficacy and safety. Relatively mild complications and ease of mastery also make TA the first choice. For properly trained medical professionals, TS is a better alternative to TA in some situations, such as APs localized in LPL or LPS, patients with aortic lesions, and failure of TA. Further research is needed to realize zero-fluoroscopy atrial septal puncture and to simplify this technology.

## Data Availability Statement

The raw data supporting the conclusions of this article will be made available by the authors, without undue reservation.

## Ethics Statement

The studies involving human participants were reviewed and approved by Beijing Children’s Hospital. The patients/participants provided their written informed consent to participate in this study.

## Author Contributions

XY and ZD: first authors, date curation, formal analysis, investigation, visualization, and writing – original draft. LG, WS, LL, and LC: resources. WY and ZZ: validation and writing – review and editing. YY: conceptualization, writing – review and editing, and funding acquisition. All authors contributed to the article and approved the submitted version.

## Conflict of Interest

The authors declare that the research was conducted in the absence of any commercial or financial relationships that could be construed as a potential conflict of interest.

## Publisher’s Note

All claims expressed in this article are solely those of the authors and do not necessarily represent those of their affiliated organizations, or those of the publisher, the editors and the reviewers. Any product that may be evaluated in this article, or claim that may be made by its manufacturer, is not guaranteed or endorsed by the publisher.
